# Does proximity to physical activity infrastructures predict maintenance of organized and unorganized physical activities in youth?

**DOI:** 10.1016/j.pmedr.2015.09.005

**Published:** 2015-09-21

**Authors:** Jason MacKenzie, Jennifer Brunet, Jonathan Boudreau, Horia-Daniel Iancu, Mathieu Bélanger

**Affiliations:** a Faculty of Medicine and Health Sciences, Université de Sherbrooke, 18 avenue Antonine-Maillet, Moncton, NB E1A 3E9, Canada; b School of Human Kinetics, Faculty of Health Sciences, University of Ottawa, 125 University Pr., Montpetit Hall 339, Ottawa, ON K1N 6 N5, Canada; c Centre de formation médicale du Nouveau-Brunswick, 18 avenue Antonine-Maillet, Moncton, NB E1A 3E9, Canada; d École de kinésiologie et du loisir, Faculté des sciences de la santé et des services communautaire, Université de Moncton, 18 avenue Antonine-Maillet, Moncton, NB E1A 3E9, Canada; e Dr George L. Dumont University Hospital Centre, Vitalité Health Network, 330 Université Avenue, Moncton, NB E1C 2Z3, Canada

**Keywords:** Organized physical activity, Unorganized physical activity, Physical environment, Recreational infrastructures, Adolescents

## Abstract

Physical activity (PA) infrastructures can provide youth chances to engage in PA. As determinants of organized and unorganized PA (OPA and UPA) may differ, we investigated if proximity to PA infrastructures (proximity) was associated with maintenance of OPA and UPA over 3 years.

Youth from New Brunswick, Canada (n = 187; 10–12 years at baseline) reported participation in OPA and UPA every 4 months from 2011 to 2014 as part of the MATCH study. Proximity data were drawn from parent's questionnaires. Proximity scores were divided into tertiles. Kaplan–Meier and Cox proportional hazard models were used to assess associations between proximity and maintenance of OPA and UPA.

There were no crude or adjusted differences in average maintenance of participation in OPA [mean number of survey cycle participation (95%CI) was 6.6 (5.7–7.5), 6.3 (5.5–7.1), and 5.8 (5.1–6.6)] or UPA [6.8 (6.2–7.4), 5.9 (5.3–6.5), and 6.6 (5.9–7.3)] across low, moderate, and high tertiles of proximity, respectively.

Findings suggest that proximity does not affect maintenance of participation in OPA or UPA during adolescence. Other environmental aspects may have a greater effect. Further research is needed before conclusions can be made.

## Highlights

• Proximity to PA infrastructures was not related to participation in OPA.
• Proximity to PA infrastructures was not related to participation in UPA.
• Youth living in rural neighborhoods maintained more OPA and UPA.

## Introduction

High levels of physical inactivity are a worldwide problem (Lim et al., 2012). In Canada, only 7% of boys and girls aged 6–19 years meet the national physical activity (PA) guidelines of 60 minutes of moderate to vigorous PA (MVPA) per day (Colley et al., 2011). In general, PA levels peak around the age of 11 years and then decrease rapidly during adolescence (Colley et al., 2011, Kahn et al., 2008, Troiano et al., 2008). Low levels of PA are associated with an increased risk of developing non-communicable diseases, including coronary heart disease, type 2 diabetes, and certain cancers (Lee et al., 2012).

PA is a complex, multidimensional behavior that is influenced by intrapersonal, interpersonal, environmental, and political factors (Glanz et al., 2008). Given theoretical tenets and research data suggesting that the physical environment affects PA by influencing both intrapersonal and interpersonal aspects of the behavior (Giles-Corti and Donovan, 2002, Stokols, 1996), the physical environment has become an important target for research and intervention (Aarts et al., 2010, Charreire et al., 2012, Haerens et al., 2009, Sugiyama et al., 2010, Wilson et al., 2012, Winters et al., 2010). Closer proximity to points of interests such as parks and trails is one component of the physical environment that may promote participation in PA (McCormack et al., 2008). For example, living within .75 miles from a commercial multi-purpose exercise facility is positively associated with PA behavior among grade 12 girls (Dowda et al., 2009). Similarly, shorter perceived distance to public and private recreational facilities is positively linked to increased PA behavior in adolescents (Ries et al., 2011). Furthermore, better land-use mix within the neighborhood is a facilitator of walking and MVPA in high school students (Voorhees et al., 2011). This is likely because adolescents who have access to various activity sites are more likely to be active at these sites (Grow et al., 2008).

Although these studies provide evidence that adolescents' PA behavior could be fostered by making environmental changes, the main outcome of these studies has been PA at a particular time rather than maintenance in PA (Boone-Heinonen et al., 2010b, Frank et al., 2007, Rosenberg et al., 2009). As one goal is to promote maintenance in PA, longitudinal research is needed to determine if the environment influences adolescents' PA levels over time, particularly during a period marked by a steep decline in PA (Colley et al., 2011), to confirm that making changes at this level would result in sustained behavior. In addition, the type of PA has rarely been considered in these studies. Although PA encompasses a wide variety of behaviors, including occupational activities, domestic duties, active transportation, exercise, sports, and other leisure activities (Bélanger et al., 2011), data are insufficient to determine the role the environment plays in promoting different subtypes of PA. It is plausible that the environment has a different influence on participation in different types of PA because distinct activities require various specific infrastructures and different neighborhoods may provide higher or lower exposure to infrastructures that facilitate certain types of PA. Thus, further research is needed to understand if the physical environment, namely, proximity to PA infrastructures, is differentially associated with certain types of PA.

The wider literature on PA suggests that PA can be categorized as unorganized or organized PA (Hardy et al., 2014). Organized physical activities (OPA) tend to require a coach or an instructor, are structured, and require payment (Bengoechea et al., 2010). Participation in OPA has been shown to be positively associated with proficiency of fundamental movements, physical fitness, and self-esteem, and negatively associated with obesity, future depressive symptoms, and risky health behaviors (Brunet et al., 2013, Hardy et al., 2014, Pate et al., 2000, Tremblay and Williams, 2003, Tremblay et al., 2000). Unorganized physical activities (UPA), in comparison, are more often practiced in a free-play manner with limited rules and without a coach or instructor (Bengoechea et al., 2010). Similar to OPA, participation in UPA is positively associated with adolescent physical fitness and the development of fundamental movement skills (Hardy et al., 2014). UPA is also associated with the development of creativity; imagination; dexterity; physical, emotional, and cognitive strength; and reduced psychological distress (Ginsburg, 2007, Haugen et al., 2014).

While studies have shown that both OPA and UPA decrease with age (Bélanger et al., 2009, Hardy et al., 2014, Stubbe et al., 2005), participation in UPA tends to decrease at a higher rate than OPA (Hardy et al., 2014). One plausible reason for this is that the environment may be more conducive to the promotion of OPA in later adolescence. Studies have demonstrated that participation in afterschool OPA is influenced by the number of sports offered in schools and that access to PA equipment is positively associated with UPA (Haerens et al., 2009, Pabayo et al., 2006). High schools may present greater opportunities for OPA by offering many opportunities to be part of sports teams, but little opportunity to use infrastructures for UPA. Similarly, environmental characteristics around the home may be differentially associated with OPA and UPA (Haerens et al., 2009, Heitzler et al., 2006). Studies have shown that having a variety of play areas around the home is associated with UPA, whereas having multiple opportunities to practice PA is associated with OPA (Haerens et al., 2009, Heitzler et al., 2006). In an effort to better understand the relationships between the physical environment and specific types of PA, we explored the associations between proximity to PA infrastructures and the maintenance of participation in OPA and UPA among children entering adolescence.

## Methodology

### Participants

We used a subsample of participants drawn from the Measuring Activities of Teenagers to Comprehend their Habits (MATCH) study, an ongoing prospective study of youth recruited from grade 5 and 6 classes in 17 schools across the province of New Brunswick, Canada. The full study protocol is described elsewhere (Bélanger et al., 2013). Briefly, 802 youth (51% of those eligible) provided parental consent and participant assent to participate in the MATCH study in the first year of data collection (2011), after ethics approval was obtained. During regular class time and under supervision of trained research assistants, participants completed a self-report questionnaire three times per year (approximately every 4 months) to assess demographic characteristics, MVPA, and types of PA practiced. At the time of running the analyses, MATCH study data were available for nine cycles collected over 3 years.

Information on environmental characteristics pertaining to PA were collected from one parent/guardian (72.9% mothers) by phone in 2011–12 using a standardized questionnaire. Contact information was available for 490 parents whom we attempted to contact a minimum of three times at various times throughout the day. We were able to reach and collect information from 187 parents. Our analyses were limited to participants for whom we had self- and parent/guardian-reported data (n = 187). Of note, these participants reported similar average weekly PA levels than those not included in the analysis based on ANOVA test (p = 0.42, 0.82, and 0.97 for years 1, 2, and 3, respectively)

### Dependent variables

At each of the nine survey cycles, participants reported all free-time PA in the past 4 months using a list of 36 activities. This questionnaire is similar to other PA checklists validated among youth (Crocker et al., 1997, Janz et al., 2008, Sallis et al., 1993) and was designed to include PA commonly engaged in by youth in Atlantic Canada (Craig et al., 2001). Using response options including “never,” “once per month or less,” “2–3 times per month,” “once per week,” “2–3 times per week,” “4–5 times per week,” and “almost every day,” participants reported (i) how often outside their gym class and (ii) with whom (i.e., alone, organized group or team, siblings, friends, parents) they most often practiced each activity. PA during gym classes were excluded because youth do not have control over activities executed in the context of these classes. Seven activities were classified as UPA, regardless of whom they were performed with (i.e., home exercises, trampoline, games, skipping rope, weight lifting, indoor chores, and outdoor chores). Although activities such as trampoline and jump rope can take place in an organized setting in some regions, the categorization of these activities as non-organized in this study was based on knowledge that they are not available in an organized setting in or around the communities from which we sampled participants. The remaining 29 activities were also categorized as UPA only if participants reported taking part in the activity by him/herself, with siblings, friends, or parents. Otherwise, if participants reported involvement in the activity with an organized group or team, these 29 activities were classified as OPA. Initial participation in both categories was defined “yes” if participants reported taking part in one or more activity at least once per week at each of the first three survey cycles. Maintenance of participation in both PA categories was assessed by verifying that participants took part in one or more activity within the respective categories at least once per week at each of the following six survey cycles.

Prior to our main analyses, we assessed the internal validity of our classification of activities into OPA and UPA categories by performing a second order exploratory factor analysis (EFA) using the FACTOR procedure in SAS software (SAS Institute Inc, Cary, NC). For this, we followed three steps outlined by Gorsuch (2008). We began by performing a first-order EFA (Model 1) using the maximum likelihood (ML) estimation method, with squared multiple correlation (SMC) prior communality estimates, and promax (power = 3) rotation was used to extract primary factors in accordance to the proportion criterion (Gorsuch, 2008). Given that the frequency of participation in each of the activities in the checklist is defined as ordinal variables and have distributions that violate the assumptions of normality, the EFA was performed on a Spearman rank correlation matrix (Gorsuch, 2008). In this first step, we retained 11 factors, most of which were homogeneous with respect to our pre-defined OPA and UPA categories. This model was deemed appropriate based on goodness-of-fit criteria (SRMR = 0.026) and the original principles of simple structure proposed by Thurstone (as cited by Gorsuch, 2008). We then executed a second-order EFA (Model 2) using ML estimation, SMC prior communality estimates, and quartimax rotation on scores computed from the primary factors (from Model 1) to further reduce the number of factors. Model 2, representing a two-factor solution, presented an improvement in goodness of fit (SRMR = 0.068) and was retained over Model 1. Third, the higher order factors in Model 2 were expressed as functions of the original variables though multiplication of the Model 1 and Model 2 factor pattern matrices (Gorsuch, 2008). Examination of the orthogonal pattern matrix (loadings) revealed that the first factor in Model 2 followed our proposed OPA and UPA classification. When communalities are low, such as in the present case (max = 0.439), there is a large propensity to capitalize on the chance characteristics of the data, resulting in non-generalizable extraneous factors (Gorsuch, 2008). The low proportion of explained common variance (0.156) and large number of cross-loaded variables (8 out of 11 salient variables) for the second factor in Model 2 suggested that it should be dropped in favor of the more parsimonious one factor model (Model 3). The one-factor model (Model 3) had evidence of marginal goodness of fit (SRMR = 0.081) (Hu and Bentler, 1999). Using a salience criterion of 0.3, there was a high correlation between salience and OPA (phi = − 0.84528), indicating correspondence between the proposed classification and the model derived from the data. This provides empirical support for the dichotomous classification of variables used herein.

### Independent variable

Data on proximity to PA infrastructures were collected using the “proximity to recreation facilities” subscale of the Neighborhood Environmental Walkability Scale for Youth (NEWS-Y) (Rosenberg et al., 2009). Parents/guardians were asked, “About how long would it take you to walk (on your own, without your children) from your home to the nearest recreation place listed below? Please indicate the time it would take you to walk to each place, even if you don’t normally go there.” This was followed by a list of 14 points of interests that can be linked to PA, such as walking trails, small public parks, and indoor exercise facilities. Response options were “1 to 5 minutes,” “6 to 10 minutes,” “11 to 20 minutes,” “21 to 30 minutes,” “31 minutes or more,” and “don't know.” Responses for each item were scored 5, 4, 3, 2, 1, and 0, respectively, and then summed as suggested by Rosenberg et al. (2009). Scores ranged from 14 to 70, with higher scores representing closer proximity to PA infrastructures. Cronbach's alpha and intraclass correlation coefficient values for scores on this subscale of .83 and .67, respectively, have been reported when completed by parents of adolescents (Rosenberg et al., 2009). For our analyses, proximity scores were divided into tertiles such that participants whose scores were between 14 and 28 were considered to be living in low proximity to PA infrastructures, the second tertile included scores of 29–40, and participants reporting scores higher than 40 represented the high proximity tertile.

### Potential covariates

Information on participants' sex was self-reported. Information on household income and both parents' educational attainment were obtained from the parent questionnaire. Given the colinearity between household income and parents' educational status, and similarity in results based on either variable, we only present results linked to parental educational status (i.e., “none” or “one or both parents had a university degree”). Rural or urban status was obtained based on postal codes reported by participants. Postal codes were entered into the address finder function on the Post Canada website to find participants' municipality of residence. Participants were considered to live in a rural setting if the municipality of residence was populated with less than 10,000 residents or in an urban setting if it included 10,000 residents or more (CID-BDC, 2011).

### Data analysis

The percentage of participants who reported participation in each of the physical activity categories in each year of follow-up was examined in repeat cross-sectional analyses. Trends in the prevalence of participation over 3 years were assessed with two-sided Cochrane–Armitage tests for trend. Kaplan–Meier analyses were performed to assess the univariate associations between proximity to PA infrastructures and maintenance of participation in OPA and UPA. Cox proportional hazard models were then used to assess the same associations while accounting for potentially confounding variables (sex, parental education, urban–rural status). Given our sample includes three pairs of siblings; we ran analyses with and without these six participants. Results for these two series of analyses were identical so only results including all participants are presented. Trend tests were computed using SAS, version 9.4, and survival analyses were performed using IBM SPSS, version 22.

## Results

Of the 187 participants retained for this analysis, 109 reported participating in OPA, and 60 in UPA, at each cycle of the first year of study (Table 1). Of the 64 participants in the low tertile of proximity to PA infrastructures, 21% and 67% participated in OPA and UPA during year 1, respectively. These proportions were 39% and 57% among the 62 participants with moderate proximity to PA infrastructures scores, and 26% and 51% among the 61 participants with high proximity to PA infrastructures scores. These proportions did not differ significantly across tertiles of proximity to PA infrastructures (X^2^ for OPA = 2.23, *p* = 0.4; X^2^ for UPA = 3.57, *p* = 0.2).

In repeat cross-sectional analyses (Table 2), the prevalence of participation in OPA, UPA, and both UPA and OPA was consistently higher during the first year of measurement than in the second and third years. The decline in rates of cessation of participation over 3 years was significant for each of these three physical activity categories. Most of the participants who reported taking part in UPA also engaged in OPA. In our sample, the prevalence of non-participation in either OPA or UPA increased significantly over time, resulting in approximately half of the participants not taking part in either OPA or UPA in the last year of follow-up.

Participation in OPA and UPA was poorly maintained throughout all survey cycles as only 20% and 25% of participants who initially reported participating in OPA and UPA, respectively, continued to do so for 3 years. Based on Kaplan–Meier analyses, tertiles of proximity were not related to PA maintenance for either OPA or UPA (Table 3). Kaplan–Meier analyses revealed that participants who lived in a rural neighborhood were more likely to maintain their participation in UPA than those who lived in an urban neighborhood. No other independent variables were significantly associated with maintenance of OPA or UPA in univariate analyses.

Based on the Cox proportional hazard models which included potential confounders, proximity to PA infrastructures was not significantly associated with maintenance of participation in either OPA or UPA (Table 4). Living in a rural setting remained a significant predictor of maintenance of participation in OPA and UPA.

## Discussion

Previous cross-sectional studies have demonstrated a positive relationship between proximity to recreational infrastructures and PA behavior in general (Adams et al., 2009, Boone-Heinonen et al., 2010a, Rosenberg et al., 2009). The current study sought to extend these findings and examined the longitudinal associations between proximity to PA infrastructures and participation in OPA and UPA. Results do not lend support to previous findings as they suggest that proximity to PA infrastructures around youths' homes is not predictive of maintenance of OPA and UPA over a period of 3 years. Although more research is needed to confirm these findings, these results suggest that interventions aiming to support maintenance of participation in OPA and UPA during the transition between childhood and adolescence should consider targeting other potential determinants of OPA and UPA.

One possible reason why we did not find an association between proximity and OPA and UPA maintenance is that our proximity score may have been too inclusive. Previous work demonstrated that participation in certain types of PA track from adolescence to adulthood (Kjønniksen et al., 2008). Given that activity specialization could carry over into adulthood, it is possible that adolescents mainly use PA infrastructures that are in relation to their chosen activity. The score we used to measure proximity to PA infrastructure was a summary of proximity to 14 different infrastructure types. Given adolescents may use certain types of infrastructure more than others, it is possible that the score used lacked sensitivity to demonstrate a relationship between only some types of PA infrastructures and OPA or UPA.

While not the main study objective, a noteworthy finding is that living in a rural neighborhood predicted maintenance of participation in both UPA and OPA. The relationship observed for UPA is in contrast with previous studies which have generally demonstrated no relationship between urban or rural living and UPA (Sallis et al., 2000, Sandercock et al., 2010). However, it is reasonable to expect that environmental factors may influence PA participation differently when present in urban versus rural settings. For example, youth from urban neighborhoods more frequently use active transportation as a method to get to school than youth from rural communities (Carver et al., 2012, Millward and Spinney, 2011). Further, data suggest that adolescents residing in rural communities often have more positive views toward PA, are less likely to be obese, and are in better physical condition than youth living in urban areas (Chillón et al., 2011, Dancause et al., 2013, Swanson et al., 2013), which may partly explain our finding since youth who report more positive attitudes toward PA tend to be more active than youth who do not view PA as a positive experience (Deforche et al., 2006). Another potential explanation for this finding is that having a place to be active such as a large backyard has been reported as a facilitator for PA among adolescents living in rural communities (Walia and Leipert, 2012). Access to abundant green space is also commonly associated with increased PA (Boone-Heinonen et al., 2010a, Boone-Heinonen et al., 2010b). Thus, it is possible that participants who were living in rural neighborhoods had greater access to green space and therefore greater opportunity to practice certain types of PA, notably UPA, than participants who were residing in urban neighborhoods.

Our results also indicate that youth who reside in rural neighborhoods maintain OPA further into adolescence than youth who reside in urban neighborhoods. Previous studies indicated that adolescents who live in urban areas may benefit from greater exposure to PA infrastructure commonly used for OPA (Dahmann et al., 2010, Kamel et al., 2014). Although this would suggest that living in an urban neighborhood should be associated with better maintenance of participation in OPA, we observed better rates of maintenance of OPA among participants living in rural neighborhoods. This lends support to previous work which demonstrated similar results (Eime et al., 2015, Millward and Spinney, 2011). For example, a cross-sectional study of Australian residents aged 15 and over indicated that higher levels of remoteness were associated with higher levels of participation in 15 different team sports (Eime et al., 2015). One possible explanation is that residents of rural neighborhoods place greater importance on representing their region in a competitive setting due to relatively greater levels of community belonging. This would be aligned with previous findings that a higher sense of community belonging is observed among more rural communities and that there is a positive relationship between community belonging and PA level (Hystad and Carpiano, 2012, Kitchen et al., 2012). Further, since it is possible that rural dwelling adolescents must overcome greater travel distance to reach OPA infrastructures than adolescents living within city limits, their involvement in OPA may be a marker for greater dedication to their given activity, which in turn could help explain the higher likelihood of maintaining PA among these participants.

### Strength and limitations

Strengths in the present study include that participants were followed-up over 3 years, with three survey cycles per year, during a period which coincides with marked declines in PA participation (Colley et al., 2011, Troiano et al., 2008). Furthermore, our assessment of participation in OPA and UPA underwent a robust validation process and was based on reports of participation in a large number of PA to be as comprehensive as possible. However, limitations inherent to self-report questionnaires can be associated with this study. Particularly, it is possible that PA participation frequency and proximity to PA infrastructures were under- or overestimated by participants and their parents/guardians. In addition, results may not be generalizable to other populations (e.g., older adolescents, adults, youth living in different geographic areas). We also note that proximity to PA infrastructure was assessed in the first year of this 3-year study and that it is possible that the environment changed in subsequent years. Finally, our measure of proximity to PA infrastructures was based on walking distance. Future studies should account for access to motorized transportation since OPA are often practiced at a fixed location, for which vehicular transportation could be an important facilitator (Perez et al., 2011), though access to a vehicle may be negatively associated with UPA, such as walking (Steinbach et al., 2012).

In sum, this study aimed to assess associations between proximity to PA infrastructures and maintenance of OPA and UPA during the transition between childhood and adolescence. Although no such significant associations were observed over time, living in a rural neighborhood was significantly associated with greater maintenance of both OPA and UPA than living in an urban neighborhood. Given low levels of OPA and UPA maintenance, strategies and incentives to promote favorable levels of PA should be developed, tested, and implemented. Although more longitudinal studies are required to guide such interventions, our results suggest that issues that are unique to either rural or urban neighborhoods should be taken into consideration.

## Conflict of interest statement

The authors declare that there are no conflicts of interest.

## Figures and Tables

**Table 1:** Description of participants from the Measuring the Activities of Teenagers to Comprehend their Habits. (MATCH) study retained for analysis (n = 187) (New Brunswick, Canada, 2011–2014).

Variable		Frequency	Proportion of study sample
Participated in unorganized physical activity in year 1		109	58%
Participated in organized physical activity in year 1		60	32%
Proximity to PA infrastructures score	Low (14–28)	64	34%
Middle (29–40)	62	33%
High (41–68)	61	33%
Sex	Female	95	51%
Male	92	49%
Neighborhood	Rural	110	59%
Urban	77	41%
Parental education	No university degree	104	56%
≥ 1 parent with a university degree	83	44%

**Table 2:** Percentage of participation in different combination of physical activity by year of study in the Measuring the Activities of Teenagers to Comprehend their Habits (MATCH) study (New Brunswick, Canada, 2011–2014).

	Year 1 (cycles 1–3) n = 187	Year 2 (cycles 4–6) n = 162	Year 3 (cycles 7–9) n = 151	*p* (trend)^a^
Organized physical activity	58	48	45	< .001
Unorganized physical activity	32	27	26	0.02
Both unorganized and organized physical activity	27	20	20	0.007
No physical activity	37	45	49	< .001

a From two-sided Cochran–Armitage tests for trend.

**Table 3:** Univariate associations between study variables and number of survey cycles for which participation in OPA (n = 60) and UPA (n = 109) was maintained (New Brunswick, Canada, 2011–2014).

		Unorganized activities		Organized activities	
Variable		Mean number of cycles activity was maintained	95% confidence interval	Mean number of cycles activity was maintained	95% confidence interval
Proximity score	Low (14–28)	6.8	6.2–7.4	6.6	5.7–7.5
Middle (29–40)	5.9	5.3–6.5	6.3	5.5–7.1
High (41–68)	6.6	5.9–7.3	5.8	5.1–6.6
Sex	Female	6.5	6.0–7.0	6.3	5.6–7.0
Male	6.4	5.9–7.0	6.3	5.6–6.9
Neighborhood	Rural	**7.1**	**6.7–7.5**	6.8	6.1–7.4
Urban	**5.2**	**4.8–5.7**	5.6	5.0–6.2
Education	No parent with university degree	6.7	6.2–7.2	6.2	5.5–6.9
≥ 1 parent with a university degree	6.1	5.5–6.6	6.4	5.7–7.0

Bold indicates a difference that is statistically different from other category of the variable.

**Table 4:** Multivariate associations between study variables and likelihood of having maintained participation in OPA (n = 60) and UPA (n = 108) (New Brunswick, Canada, 2011–2014).

Reference group	Comparison groups	OPA			UPA		
		Exp (β)	95% CI	*p* value	Exp (β)	95% CI	*p* value
Proximity (ref: low)	Moderate	0.92	0.36–2.39	0.86	1.34	0.74–2.44	0.34
High	0.79	0.39–1.59	0.51	1.22	0.70–2.13	0.49
Sex (ref: female)	Male	0.76	0.40–1.45	0.41	0.96	0.61–1.51	0.86
Neighborhood (ref: rural)	Urban	**0.39**	**0.17–0.86**	**0.02**	**0.34**	**0.19–0.61**	**< 0.001**
Education (ref: no university degree)	≥ 1 parent with university degree	1.50	0.78–2.87	0.22	1.1	0.65–1.87	0.71

Bold indicates a difference that is statistically different from other category of the variable.
